# Tailor-made spider-eggcase-silk spheres for efficient lysosomal drug delivery†

**DOI:** 10.1039/c8ra00232k

**Published:** 2018-03-06

**Authors:** Jianming Chen, Jinlian Hu, Peijun Zuo, Xiaoqian Su, Zhigao Liu, Mo Yang

**Affiliations:** Institute of Textiles and Clothing, The Hong Kong Polytechnic University Hung Hom Kowloon Hong Kong jin-lian.hu@polyu.edu.hk; Nano and Advanced Materials Institute, The Hong Kong University of Science and Technology Clear Water Bay Kowloon Hong Kong; School of Chemical and Biomedical Engineering, Nanyang Technological University Singapore; Shenzhen PKU-HKUST Medical Center Shenzhen Guangdong China; Department of Biomedical Engineering, The Hong Kong Polytechnic University Hung Hom Kowloon Hong Kong

## Abstract

Spider silks are attractive biopolymers due to their excellent mechanical properties and biomimetic potential. To optimize the electrostatic interaction for lysosomal drug delivery, a spider-eggcase-silk protein was genetically engineered using 5× His Tag with a tailor-made isoelectric point of 4.8. By a facile HFIP-on-oil method, silk spheres were assembled as rapidly as 10 s. After the post-treatment of ethanol, silk spheres were determined with an improved compressive modulus by AFM indentation. Under incubation of silk spheres in a Doxorubicin solution, a maximum of 35% loading and average of 30% loading efficiency were determined. In the cytotoxicity experiment, silk spheres exhibited intrinsic biocompatibility and showed good control of the loaded drug in the neutral PBS solution. Significantly, by 96 h, the accumulative drug release at pH 4.5 was approximately 4.5-fold higher than that at pH 7.4. By conducting the platelet adhesion and hemolysis assay, Doxorubicin-loaded silk spheres exhibited good hemocompatibility. To further demonstrate this release behavior, within 24 h, Doxorubicin-loaded silk spheres were efficiently delivered to lysosomes and then released the payload to the nuclei of Hela cells.

## Abstract 2

Recombinant spider-eggcase-silk spheres are facilely prepared as drug carriers with a tailor-made isoelectric point specifically for lysosomal delivery.

## Graphical abstract

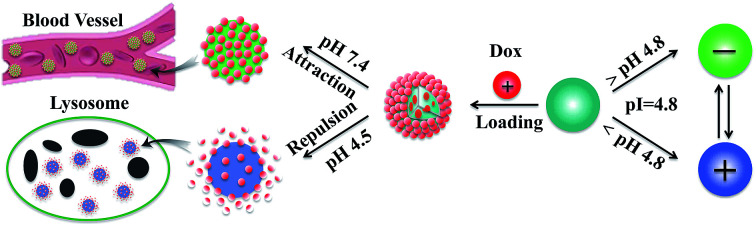

## 1. Introduction

Recent decades have seen the rapid development of spheres for cancer therapies due to their abilities in controlled size, structure, biodegradation rate, drug loading and release.^1–3^ However, a critical obstacle and challenge to the efficacy of cancer chemotherapy using spheres concerns insufficient intracellular drug release, especially in target compartments.^4^ To address this problem, strategies have been employed to build up smart delivery systems to alter the behavior of carriers under a certain stimulus.^5–7^ Of various stimuli, pH-responsiveness is favorably adopted because of wide range of pH values inside body environments. Even in the intracellular microenvironment, pH drops the endocytic pathway, from pH 6.0–6.5 in early endosomes to pH 4.5–5.5 in late endosomes and lysosomes.^8^

Cellular entry of spheres is through the endocytosis and their intracellular fate highly depends on different pathways. Even though the endocytosis mechanism still remains unclear,^9^ drug carriers often end up in lysosomes where they are subjected to acidification (typically pH = 4.5) and enzymolysis. Interestingly, polyhistidine, a well-known affinity tag for protein purification, is used to modify polymer carriers with pH-sensitivity.^10–12^ Nevertheless, limited biodegradability of these polymers leads to various side effects, such as lysosomal storage diseases.^13^ In fact, many natural biomacromolecules including spider and silkworm silks, present pH-responsiveness around the intrinsic isoelectric point (pI) through transition of net/surface charges. As studied by David Kaplan and Thomas Scheibel.^14,15^ the drug loading and release mechanism for silk spheres was mainly due to the combination of hydrophobic and electrostatic interactions. Currently, it remains challenging to evaluate the hydrophobic interplay in a systematic manner. However, a model has been put forward to explain the pH-dependent release of positively-charged Doxorubicin (Dox) based on electrostatic interactions.^16^ Therefore, it becomes feasible in choosing a suitable pI to optimize the electrostatic attraction or repulsion for Dox-loaded delivery systems.

Task-specific spider silks, assembled under different combination of proteins, enable spiders to perform several functions, including prey capture, locomotion and offspring protection in egg cases.^17^ Distinctive mechanical advantages, along with the intrinsic biocompatibility and biodegradability, make silk-based biomaterials be of considerable interest in drug delivery applications.^18^ Superior to regenerated silk fibroins, gene technology has been intensively investigated for the bottom-up control of recombinant spidroins from composition to sequence, and from structure to property. By the modification of gene codon, the positively or negatively charged amino acid residues could be easily regulated for a required pI. In spite of the great potential for silk spheres as drug carriers,^19,20^ there have been only limited studies on the silk-based lysosomal delivery,^16^ and even more rare in assessing the engineered spider-eggcase-silk protein in terms of the specific pI as a pH switch.

**Fig. 1:**
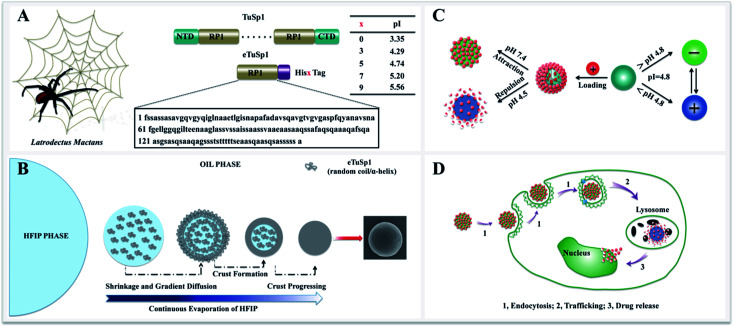
Schematic illustration of the design of eTuSp1 and the application of eTuSp1 spheres in lysosomal delivery systems. (A) Engineered spider-eggcase-silk proteins from a black widow spider. In comparison to bare proteins, the isoelectric point of recombinant proteins can be flexibly regulated from 3.35 to 5.56 by fusing with different numbers of His Tags. (B) Fabrication process of eTuSp1 spheres by the HFIP-on-oil method. (C) The pI-mediated drug loading and release of eTuSp1-Dox spheres. Silk spheres are loaded with Dox (red) in the negative (green) state and release the drug in the positive (purple) state. (D) Internalization and intracellular drug release. (1) Endocytosis; (2) trafficking; (3) drug release.

Herein, we present recombinant spider-eggcase-silk spheres with a tailor-made pI for lysosomal delivery systems. Tubuliform spidroin 1, from a black widow spider, *Latrodectuss mactans*, is engineered *via* gene recombinant and abbreviated as eTuSp1. Of various purification methods, polyhistidine-tagging is utilized as a common practice with composition ranging from 3 to 10 histidines (His) in series.^21–23^ For lysosomal delivery, ideally, the pI of silk carriers should be optimized within 4.5–5 to sufficiently retain the drug efficacy when exposed to other pH mediums. Thus, pI values of the eTuSp1 containing different numbers of His tags are first predicted by ExPASy ProtParam (Fig. 1A).^24^ Then, 5× His tags are employed in this work to purify and simultaneously modify the final eTuSp1 with a pI of 4.8, which is precisely determined by the isoelectric focusing (IEF) electrophoresis.^25^

## 2. Materials and methods

### 2.1. Gene recombinant

The gene encoding engineered eggcase silk proteins eTuSp1 were cloned, expressed and then purified (Fig. S1†). TuSp1, from the black widow spider, *Latrodectuss mactans*, was taken as the template, in which the repetitive domain was composed of a single conserved repeat unit. Thus, single conserved oligonucleotide, encoded from the consensus motifs of TuSp1, was engineered with His5 Tag. The resultant proteins had a molecular mass of 15 kDa. Prior to use, proteins were lyophilized and stored at −20 °C. Subsequently, the protein solution was prepared in HFIP (1,1,1,3,3,3-hexafluoro-2-propanol) and adjusted to the required concentrations, determined by UV adsorption method.

In the stage of cloning, DNA construct was designed to produce truncated TuSp1. The eTuSp1 construct was engineered with a single repetitive unit for the following prokaryotic expression. To amplify the target cDNA, PCR with 7 forward and 7 reverse oligonucleotides were assembled into the eTuSp1 gene. The gene was optimized with condon usage table to maximize the expression. The eTuSp1 was amplified in accordance to a previously characterized TuSp1 cDNA with residues 1-161 (GenBank: AAY28947.1). Following amplification, the cDNA fragment was gel extracted and ligated into the plasmid vector pUC18-SN and subsequently transformed into *E. coli*. Restriction enzyme digestion and agarose gel electrophoresis were conducted to validate if the cDNA was inserted in the correct direction. Upon verification, the modified plasmid with the cDNA carried was subjected to the DNA sequence analysis.

For protein induction, a 200 mL of LB was used as 10% inoculum. At midlog phase of *A*_600_ = 0.6–1, 50 μg mL^−1^ ampicillin was added and the protein was induced with 2 mL isopropyl-β-d-thiogalactoside (IPTG) at 37 °C for 2 h. Following induction, the bacterial cells were lysed under sonication for 20 min (10 s/10 s) using a Bandelin SONOPULS ultrasonic homogenizer. After sonication, the sample was clarified at 6000*g* for 5 min. The obtained supernatant was examined by Coomassie blue staining and immunoblotting. The protein was then combined with Ni-NTA agarose resin *via* the engineered His-tag with the purification experiment performed according to the manufacturer's instructions. Finally, the purified protein was determined by SDS-PAGE to identify the protein expressed by the clones.

### 2.2. Spheres formation

8 μL silicon oil, hydroxyl terminated polydimethylsiloxane (*M*_w_ = 4200, International Laboratory USA) was pipetted onto a clean silicon wafer. A drop of 2 μL spidroin solution was injected to the oil with a rapid assembly as short as 10 s. Accompanied by the evaporation of HFIP, silk spheres were formed with concentrations ranging from 0.5 to 10 mg mL^−1^. Embedded in oil phase, the fresh-assembled spheres were observed by optical microscopy. To induce β-sheets formation for the structural stabilization, spheres were transferred from the oil phase into the aqueous phase by re-suspension in the methanol/water (60/40) mixture solution, followed by three washing steps with water. Finally, silk spheres can be prepared with the size ranging from 150 nm to 35 μm.

### 2.3. Drug loading and release

#### 2.3.1 Loading process

Loading with Doxorubicin (Sigma-Aldrich) was conducted in 10 mM phosphate buffer at pH 7.4. The eTuSp1 spheres suspension and Doxorubicin (Dox) solution was mixed to obtain the desired w/w-ratio (%) of Dox to silk spheres (0.5 mg mL^−1^). This loading procedure lasted for 1 h at room temperature under gentle agitation. The Dox-loaded eTuSp1 (eTuSp1-Dox) spheres were obtained after centrifugation at 12 000*g*, 40 min. The amount of Dox loaded into the spheres was calculated spectrophotometrically by measuring the absorbance of Dox at a wavelength of 509 nm. A standard calibration curve for the Dox (from 0.25–100 μg mL^−1^) is combined with the control groups to quantify the loading and loading efficient.

#### 2.3.2 *In vitro* release behaviors

All the experiments were performed with the eTuSp1-Dox spheres of 25% payload, incubated at 37 °C under constant shaking. The solvent was periodically removed and replaced with fresh medium. The release drug removed from the medium was determined by UV-Vis-spectrometry. Finally, the release of eTuSp1-Dox spheres at three pH values was studied as a function of incubation time.

#### 2.3.3 *In vitro* cytotoxicity studies

Prior to use, Hela cells provided by Prof. Mo Yang were cultured in the 5% CO_2_ at 37 °C. The toxicity of Dox, eTuSp1 and eTuSp1-Dox spheres was investigated by plating 1 × 10^5^ cells per mL in 96-well plates. The cell viability was determined with a three-day incubation in the PBS solution by using 3-(4,5-dimethylthiazol-2-yl)-2,5-diphenyltetrazolium bromide (MTT).

### 2.4. Hemocompatibility

The plate-rich plasma (PRP) and red blood cells (RBCs) were prepared as reported elsewhere.^26,27^ Briefly, fresh blood from the healthy volunteer was stabilized with heparin and then centrifuged at 2000 rpm for 15 min to separate the plasma and precipitation. All experiments were performed in compliance with the guidelines of the Human Subjects Ethics Sub-Committee (HSESC) and approved by the ethics committee at the Hong Kong Polytechnic University. Informed consents were obtained from human participants of this study.

Platelet adhesion assay: the plasma was diluted 10 times in PBS buffer and 200 μL platelet/heparin plasma was added into 800 μL of each sample for 120 min incubation at 37 °C. The pure PBS buffer (pH 7.4) was taken as control. Dynamic light scattering (Zetasizer Nano ZS) was used to determine the size of silk spheres in the different medium, thus indicating their stability in blood and affinity to platelet.

Hemolysis assay: the precipitation was thoroughly rinsed with PBS buffer (pH 7.4). By diluting 10 times with PBS buffer, 200 μL RBCs suspension was added into 800 μL of each sample for 2 h incubation at 37 °C under gentle shaking. Pure PBS and 1% Triton X100 was repectively used as negative and positive control, allowing the calculation of hemolysis percentage. After centrifugation of samples at 8000 rpm for 5 min, the absorbance of hemoglobin in the supernatant can be recorded by UV-vis spectrophotometer (Ultrospec 2100 Pro) at 545 nm. Each sample and control was repeated for 3 times.

### 2.5. Method and characterization

#### 2.5.1 Analytical isoelectric focusing (IEF)

Isoelectric focusing was carried out in the MODEL 111 MINI IEF CELL (Bio-Rad) by using 4% nondenaturing polyacrylamide gels with ampholytes in a pH range from 3 to 10 at 4 °C. And the pI standards were also obtained from Bio-Rad. The pI of the eTuSp1 was determined according to the manufacturer.

#### 2.5.2 Zeta-potential

The zeta-potential of eTuSp1-Dox spheres was determined with Zetasizer Nano ZS (Malvern Instruments, UK) at 37 °C under the same concentration.

#### 2.5.3 DLS

The size and polydispersity index of the eTuSp1 spheres was determined by dynamic light scattering measurement conducted with Zetasizer Nano ZS. All samples were tested at 25 °C under the same concentration.

#### 2.5.4 ATR-FTIR

The confirmation identification of silk spheres with/without methanol treatment was recorded on Nicolet 5DXC. Samples were directly deposited on the ATR crystal and the measurement was performed after the evaporation of solvent.

#### 2.5.5 FE-SEM

FE-SEM (JEOL JSM-6335F) was employed to determine surface morphology of silk spheres. The spheres solution was pipetted onto a conductive silicon wafer and allowed to dry in the air overnight. Then, the dried samples were coated with platinum/palladium for 90 s with an ion sputter, followed by the operation at a voltage of 15 kV.

#### 2.5.6 TEM

The diluted solution of HFIP-dissolved eTuSp1 and eTuSp1 spheres was added dropwise to an electron microscopy specimen grid covered with a thin carbon support. TEM micrographs were recorded on a JEOL JEM-2100F operating at a voltage of 200 kV.

#### 2.5.7 AFM

AFM (Bruker Nanoscope SPM8) was utilized with an ACTA-20 silicon probe. The pyramid-tipped cantilever was determined by the thermal tune method with an average spring constant *k* of 8.90 ± 0.11 N m^−1^. The topographic images and force–distance curves were respectively recorded by the mapping mode and indent mode under ambient conditions (20 °C and 50% relative humidity). Silicon wafer was cleaned by sonication with acetone, ethanol and DI water, followed by plasma treatment for 1 min. Silk spheres were allowed to settle on the clean silicon wafer, and the cantilever was positioned over a single sphere. By assuming a Poisson ratio of 0.5, compressive modulus of spheres was calculated using Hertz model.

#### 2.5.8 CLSM

Confocal images of the silk spheres were performed on a Leica TCS SP8 system with a 63 × 1.4 oil immersion objective lens. The Hela cells were incubated in a 5% CO_2_ atmosphere for 24 h with the complete DMEM at 37 °C. The cells were stained by Hoechst 33 342 (Sigma Aldrich) for cell nucleus and LysoTracker Green (Thermo Fisher Scientific) for lysosomes following the manufacturer's instructions. The eTuSp1-Dox spheres with 25% payload were used for subcellular distribution observation. Before adding the eTuSp1-Dox spheres into the culture medium, they were first put in the fresh PBS solution for 24 h and then centrifuge in 6000*g* for 15 min. After incubation for 2 h, the culture medium was washed twice by PBS solution and replaced by fresh medium.

## 3. Results and discussion

With eTuSp1 dissolved in HFIP, eTuSp1 spheres were assembled as rapidly as 10 s by a facile HFIP-on-oil method (Fig. 1B). Distinct from the traditional water-in-oil emulsification, eTuSp1 solution was not immersed into the oil medium but spread on the oil surface (Fig. S2†). In this system, a variety of factors, including the viscosity, surface tension and solvent volatilization significantly affect the formation of silk spheres. To be specific, eTuSp1 solution was first spreading on the oil surface and then was split into tiny droplets due to the interfacial tension of HFIP–oil–air. Accompanied by fast evaporation of HFIP, a silk crust formed and continued to grow until all the solvent was completely transported out. Finally, silk spheres were obtained from the liquid to solid phase separation. It is worth noting that the size and shape of resulting eTuSp1 spheres are largely defined by the premature droplets, which was similarly reported in the emulsification method.^15^

The charge transition of an eTuSp1 sphere occurs in response to a pH above or below its pI (Fig. 1C). It is assumed that, eTuSp1 spheres with the pI of 4.8 are negatively charged in the extracellular environment, like blood plasma (pH 7.4), but become positively charged in the intracellular environment, like lysosomes (pH 4.5). Therefore, the pI of eTuSp1 spheres may act like a pH switch capable of controlling the positive drug such as Dox by electrostatic interactions. After internalized by cancer cells, the payload can be released by the trigger of lysosomal pH (Fig. 1D).

**Fig. 2:**
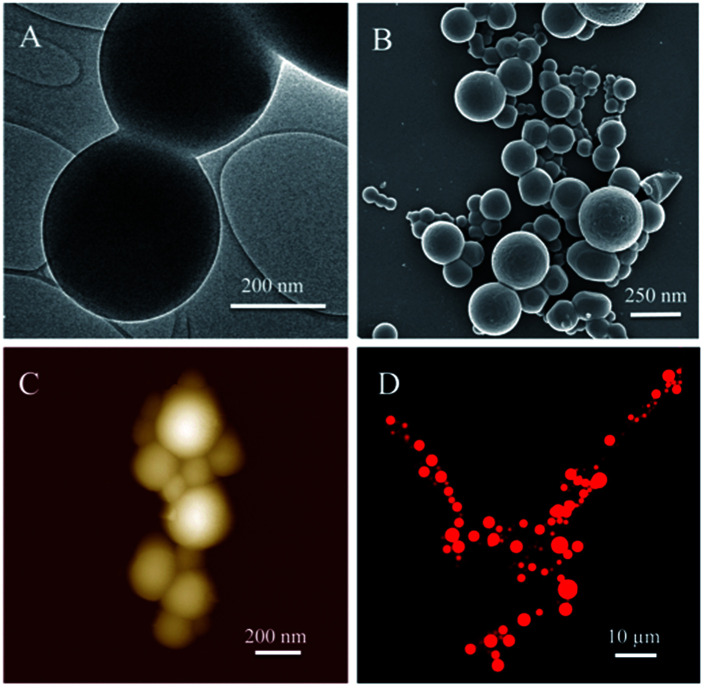
The morphology structure of silk spheres and their potential in drug loading. (A) TEM image of compact eTuSp1 spheres. (B) FE-SEM image of dried eTuSp1 spheres. (C) AFM image of eTuSp1 spheres. (D) CLSM image of eTuSp1-Dox spheres.

TEM results exhibited the inner structure of eTuSp1 spheres (Fig. 2A). The spheres were observed with solid, compact and uniform structures. Apparent in the FE-SEM image (Fig. 2B), silk spheres had a dense surface free of visible defects, which was further revealed by AFM (Fig. 2C). Micrometer-sized spheres were prepared to investigate the loading property by the confocal laser scanning microscopy (CLSM). The CLSM image of eTuSp1-Dox spheres demonstrated that the Dox not only adhered to the surface but also diffused into the matrix (Fig. 2D).

**Fig. 3:**
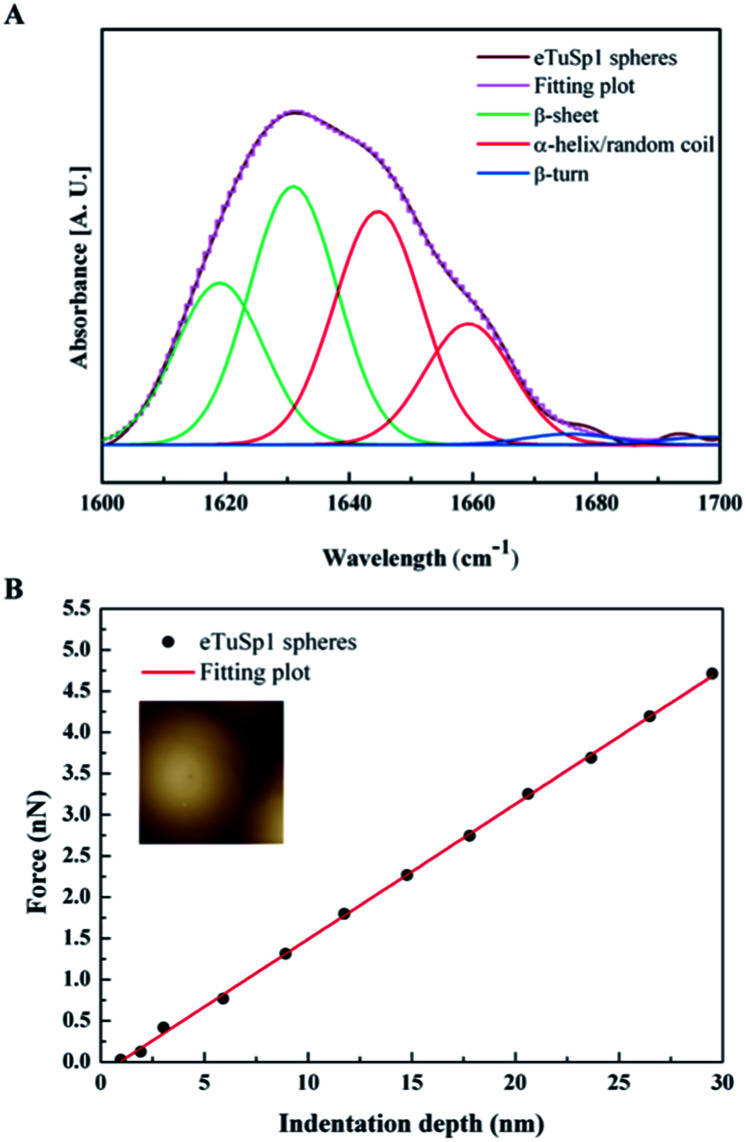
The secondary structure and compressive modulus of eTuSp1 spheres. (A) ATR-FTIR spectra of eTuSp1 spheres. (B) Mechanical property of eTuSp1 spheres. The compressive force is investigated as a function of indentation depth. The inset shows the AFM image recorded for the local force measurement on a single sphere.

In the post-treatment stage, eTuSp1 spheres were conducted with ethanol solution for improved structure integrity. The stability of treated silk spheres in PBS solutions (pH 7.4) lasted more than 4 days. As shown in Fig. 3A, the secondary structure of eTuSp1 spheres was investigated by ATR-FTIR spectroscopy. The β-sheet-rich eTuSp1 spheres were confirmed with a major peak centering on 1634 cm^−1^ in comparison to amorphous original ones (Fig. S3†). Deduced by Fourier self-deconvolution, the contribution of each peak at 1619, 1631, 1645, 1659, 1676 and 1698 cm^−1^ was presented, respectively. These peaks were correspondingly assigned to β-sheets, random coil/α-helix and β-turns. Notably, substantial β-sheets within eTuSp1 spheres were detected up to 53%, comparable to those in natural spider eggcase silks (∼46%).^28^

A number of studies have shown that size, shape and surface chemistry have tremendous impacts on cellular internalization of nanocarriers.^9,29–32^ Thus we evaluate the anti-deformation properties of silk spheres with the aim at maintaining their spherical shape before reaching the target site. Mechanical properties of eTuSp1 spheres were investigated by AFM indentation (Fig. 3B). While, the deformation of individual eTuSp1 spheres was conducted by measuring the force–displacement curve (Fig. S4†). According to the Hertz model, when applied with a load *F*, the deformation, *h*, of the sphere is given by:^33^1
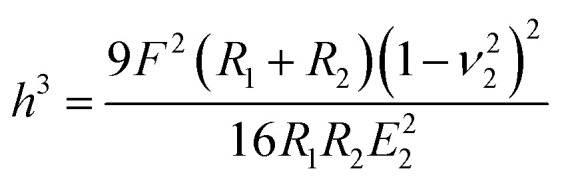


Then, the compressive modulus is calculated as:2
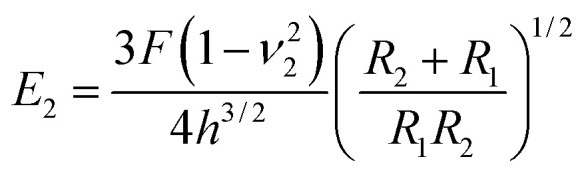
where *ν*_2_ is the Poisson ratio of the sphere, *R*_1_ and *R*_2_ is the radius of AFM tip and the sphere, respectively. In our system, the radius of AFM tip is known as 6 nm and individual spheres of ∼183 nm are selected for measurement. With the assumption of a Poisson ratio *ν*_2_ of 0.5, eTuSp1 spheres were determined to have a modulus of 9.6 ± 2.8 MPa. This value reflected a strong characteristic, considering the reported modulus of silkworm silk spheres was only 1.46 ± 0.75 kPa.^34^ We attribute the mechanical property of eTuSp1 spheres to the high component of β-sheets, which are also of great significance on the following drug loading/release behaviours.

**Fig. 4:**
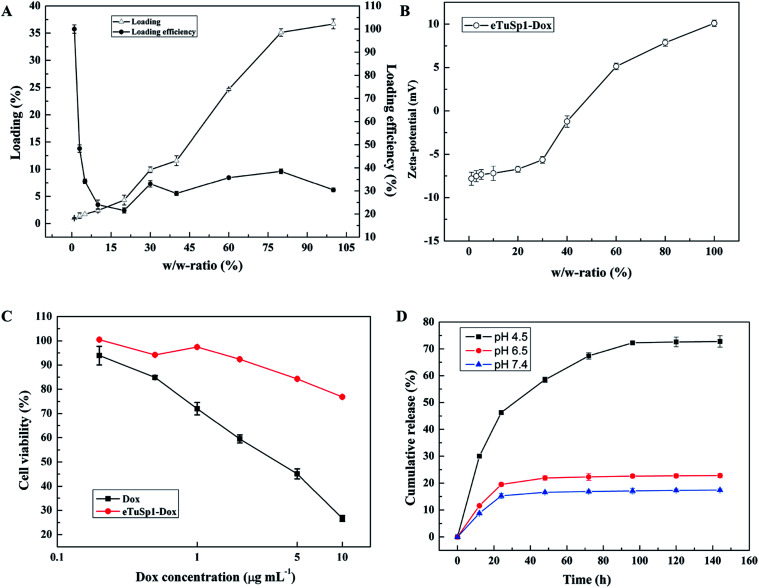
Loading and release behaviors of eTuSp1 spheres. (A) Loading and loading efficiency of eTuSp1 spheres. (B) Zeta-potential of eTuSp1-Dox spheres with regard to different w/w-ratios. (C) Cytotoxicity of free Dox and eTuSp1-Dox spheres. The Dox concentration of eTuSp1-Dox spheres is equivalent to that of the free Dox. (D) Cumulative release of eTuSp1-Dox spheres at different pH media.

In addition to the pre-loading approach, positively charged Dox can be loaded onto negative eTuSp1 spheres by charge–charge interaction in the aqueous solution. Fig. 4A shows the loading and loading efficiency of eTuSp1 spheres as a function of w/w-ratios. Simply by incubation of eTuSp1 spheres in the Dox solution, the maximum loading of Dox could be up to 35%. Except the initial stage, the loading efficiency remained around 30% at the w/w-ratios ranging from 5% to 100%, allowing a stable and controllable loading process. Loading degree plays a pivotal role on the physicochemical properties, which is evidenced by the result in Fig. 4B. Under the control of w/w-ratios (Dox to spheres), the charge of eTuSp1-Dox spheres can be regulated from negative or positive. As studied earlier, silk spheres were engineered with positive charge for an improved internalization by negative cells.^35^ Quite similar to the loading process, this charge-enhanced endocytosis is mainly attributed to the electrostatic interplay. Thus, in this work, positive eTuSp1-Dox spheres with approximately 25% payload are used to evaluate the cytocompatibility, hemocompatibility and lysosomal delivery.

The cytotoxicity of eTuSp1 spheres was examined using Hela cells at pH 7.4 (Fig. 4C). Pristine silk spheres showed rather good biocompatibility with no remarkable difference under various concentrations (Fig. S5†). Compared with the equivalent amount of free Dox, the cell viability of eTuSp1-Dox spheres was obviously improved, suggesting the effective drug retainment in this neutral environment. To demonstrate the pH-sensitive drug release, eTuSp1-Dox spheres were incubated at different pH media and the corresponding cumulative release was monitored by the standard curve of Dox concentrations (Fig. S6†).

As shown in Fig. 4D, release behaviors of eTuSp1-Dox spheres were studied over a range of mimic pH of 7.4 (blood plasma), 6.5 (early endosomes) and 4.5 (lysosomes). The release profiles of eTuSp1-Dox spheres were similar at pH 6.5 and 7.4, both of which reached the platform by 24 h with no more than 20% release of Dox. However, in a burst mode, the release of eTuSp1-Dox spheres became more rapid and substantial at pH 4.5. Although Dox obtained higher solubility in the acid solution, but its release within silk matrix was mainly dominated by electrostatic interactions.^36^ In case of Dox-loaded silk spheres reported previously,^16,37^ it was interesting to find that the release behaviors were quite similar by 24 h. To be specific, the release of Dox was about two-time higher at pH 4.5 than pH 7.4. Whereas in the present study, the release results were improved up to three times under the same condition. We attribute the difference to the improved electrostatic interactions by subtle optimization of pI on the assumption of unchanged hydrophobic interactions between Dox and silk matrix. Even more pronouncedly by 96 h, the Dox released at pH 4.5 was approximately 4.5-fold higher than that released at pH 7.4, implying the good pH sensitivity of eTuSp1-Dox spheres to lysosomes.

**Fig. 5:**
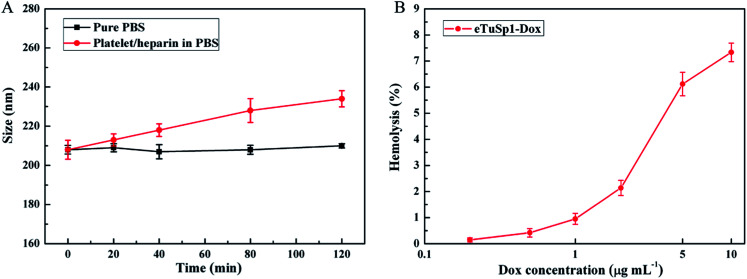
Hemocompatibility of eTuSp1-Dox spheres. (A) platelet adhesion assay. The size of eTuSp1-Dox spheres is evaluated when exposed to pure PBS and platelet/heparin solution. (B) Hemolysis assay. The hemolysis of eTuSp1-Dox spheres is conducted with Dox concentrations ranging from 0.2 to 10 μg mL^−1^.

To fulfil the envisioned function of eTuSp1-Dox spheres in blood, *in vitro* hemocompatibility was evaluated by conducting the platelet adhesion and hemolysis assay. As depicted in Fig. 5A, eTuSp1-Dox spheres were quite stable in pure PBS (pH 7.4) with limited changes in dimension. Upon exposure to the platelet/heparin solution, the size of spheres was increased with regard to the increase of incubation time. Yet, no significant dimensional change was observed (being less than 12%), partly attributing to the existence of heparin. According to previous research,^38,39^ silk-based materials can be modified by heparin so as to effectively avoid the thrombus caused by platelet adhesion. The eTuSp1-Dox spheres maintained good stability without exhibiting precipitation or phase separation in the PBS and platelet/heparin solution. Hemolysis assay was performed to monitor the release of hemoglobin caused by the disruption of erythrocyte membranes. Fig. 5B shows the hemolysis of eTuSp1-Dox spheres in the Dox concentration of 0.2, 0.5, 1, 2, 5 and 10 μg mL^−1^. The percentages of hemolytic activity in the concentration ranging from 0.2 to 2 μg mL^−1^ were less than 5% (standard acceptance limit),^40,41^ indicating excellent hemocompatibility of eTuSp1-Dox spheres. The maximum hemolytic activity percentage was observed in a Dox concentration of 10 μg mL^−1^. Therefore, in light of all these results, it is demonstrated that eTuSp1-Dox spheres show good stability in blood.

**Fig. 6:**
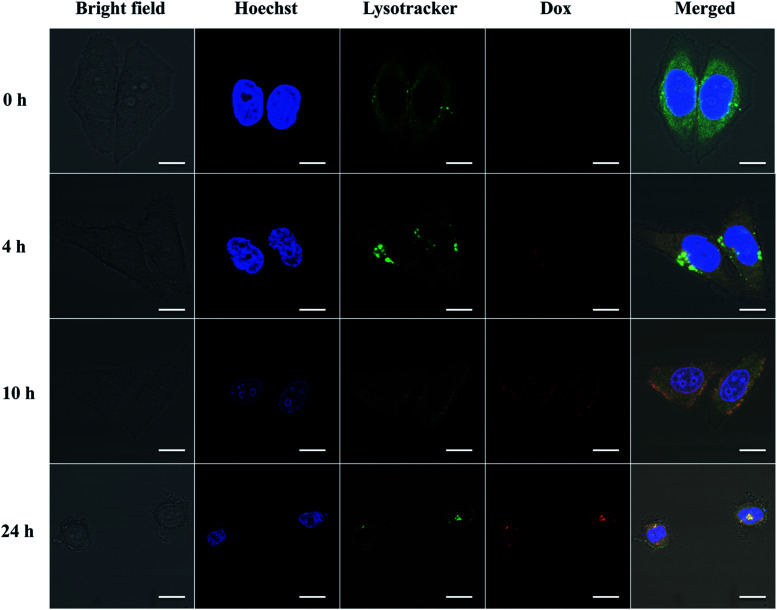
Lysosomal delivery of eTuSp1-Dox spheres in Hela cells. Hoechst and Lysotracker are used to strain the cell nucleus (blue) and lysosomes (green), respectively. The trafficking of the Dox in the intracellular environment is recorded in 24 h by the aid of fluorescence detection. The scale bar represents 10 μm.

To further demonstrate whether eTuSp1-Dox spheres could be internalized by cells and then efficiently delivered to targeted acid organelles, their intracellular fate was detected by CLSM. To date, limited work has been done to investigate the trafficking of drug-loaded silk spheres in the intracellular environment. The nuclei and lysosomes were respectively labeled with fluorescent dyes to trace the eTuSp1-Dox spheres inside Hela cells. In order to bypass lysosomes, caveolae-mediated endocytosis was often selected by viruses and bacteria to escape from enzymolysis.^42,43^ For enhanced and preferable lysosomal delivery, silk spheres were dialyzed in MQ-H_2_O with an average size of 183 nm (see Table S1†) in accordance to size-dependent endocytosis mechanism.^44,45^ Before incubation in the cell solution, eTuSp1-Dox spheres were shacked in PBS solutions to remove the loosely adhered Dox from the surface of spheres. As shown in Fig. 6, the co-localization of eTuSp1-Dox spheres and lysosomes revealed efficient lysosomal delivery, simultaneously suggesting that Dox was not released at endosomes in advance. Specifically, after 4 h incubation, eTuSp1-Dox spheres were delivered to lysosomes, as evidenced by the overlapping fluorescence. For the following 20 h, the Dox first accumulated in the nuclei and subsequently induce the corruption of tumor cells as a consequence of low pH stimulus and enzymatic degradation in lysosomes.

## 4. Conclusions

In summary, recombinant spider-eggcase-silk spheres were prepared by the HFIP-on-oil method with rapid assembly of 10 s. By fusing with 5×His tags, eTuSp1 was well purified and simultaneously obtained a tailor-made pI of 4.8 specifically for lysosomal delivery. β-sheet-rich silk spheres were determined with an average modulus of 9.6 MPa by AFM indentation. In the loading process, it was found that the loading efficiency was around 30% and the charge of eTuSp1-Dox spheres can be regulated by various loading ratios. In the MTT assay, eTuSp1 spheres showed good biocompatibility and were capable of retaining the Dox in PBS solutions (pH 7.4). Strikingly, the accumulative release of Dox by 96 h was approximately 4.5-fold higher at pH 4.5 than that at pH 7.4. According to the results of hemocompatibility assay, eTuSp1-Dox spheres exhibited good stability in blood. To further investigate this release behaviour on Hela cells, within 24 h, eTuSp1-Dox spheres were first observed to accumulate in lysosomes and then efficiently released the Dox to nuclei under the pH stimulus. The facile fabrication method and the subtle employ of pI as a pH switch establish an appealing platform to develop fascinating protein-based materials for specific drug delivery systems.

## Conflicts of interest

There are no conflicts to declare.

## Supplementary Material

RA-008-C8RA00232K-s001
