# Supplementary material for: Tailor-made spider-eggcase-silk spheres for efficient lysosomal drug delivery
Source: RSC Adv. 2018 Mar 6;8(17):9394–401. doi: 10.1039/c8ra00232k (PMC9078666; doi:10.1039/c8ra00232k)
Supplement: RA-008-C8RA00232K-s001 [file RA-008-C8RA00232K-s001.pdf]

Electronic Supplementary Material (ESI) for RSC Advances.
This journal is © The Royal Society of Chemistry 2018

Supplementary information

Tailor-Made Spider-Eggcase-Silk Spheres for Efficient Lysosomal Drug Delivery

Jianming Chen,[a] *Jinlian Hu,**[a] *Peijun Zuo,*[b] *Xiaoqian Su,*[c] *Zhigao Liu,*[d] *and Mo Yang*[e]

[a]Institute of Textiles and Clothing, The Hong Kong Polytechnic University, Hung Hom, Kowloon, Hong Kong, China

Email: jin-lian.hu@polyu.edu.hk.

[b]Nano and Advanced Materials Institute, The Hong Kong University of Science and Technology Clear Water Bay, Kowloon, Hong Kong. China

[c]School of Chemical and Biomedical Engineering, Nanyang Technological University, Singapore.

[d]Shenzhen PKU-HKUST Medical Center, Shenzhen, Guangdong, China.

[e]Department of Biomedical Engineering, The Hong Kong Polytechnic University, Hung Hom, Kowloon, Hong Kong, China

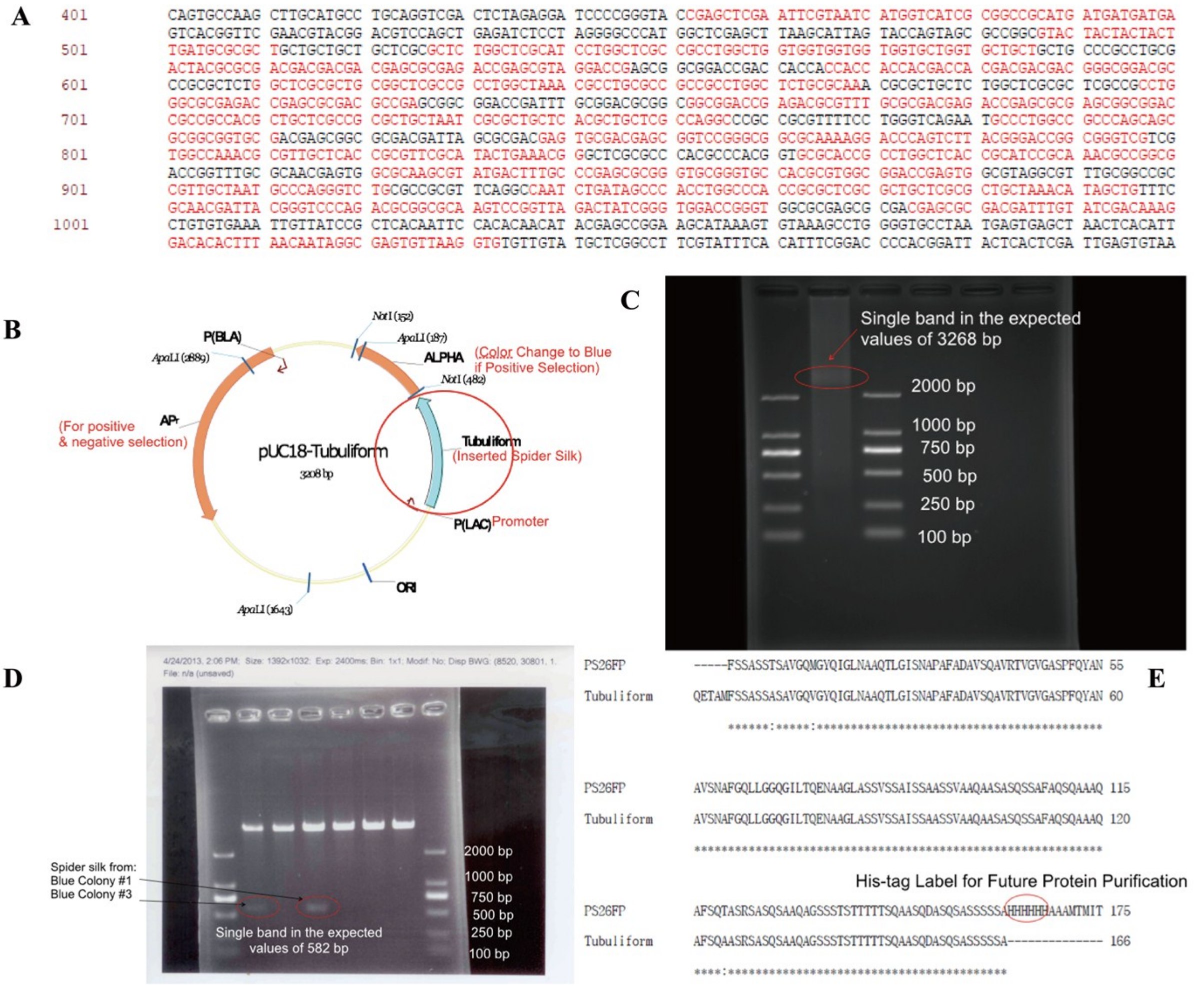

Figure S1. Gene recombinant of spider eggcase silk proteins. A, complete gene sequence; B, plasmid design; C, verification of targetted plasmid; D, verification of tubuliform spidroins; E, Similarity comparison between the wild type and engineered one.

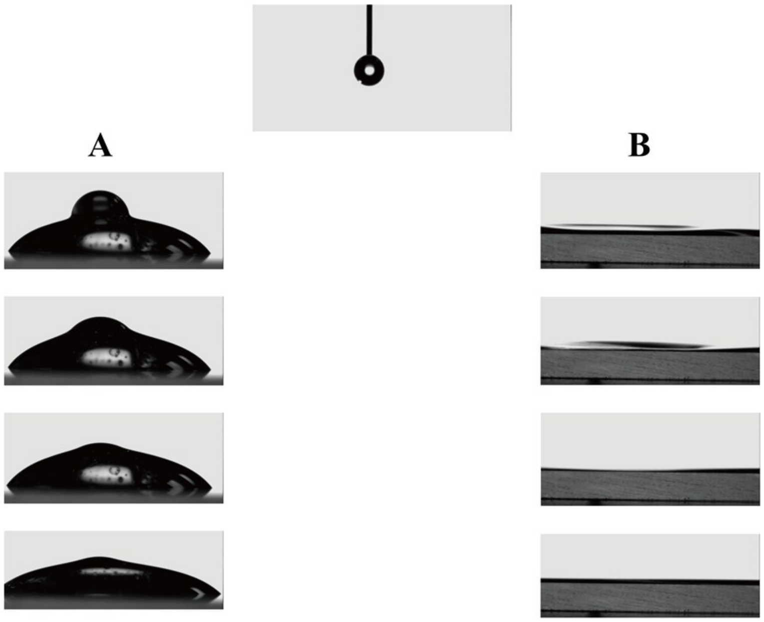

Figure S2. Preparation process in different lubricant substrates. A) High- molecule-weight silicon oil; B) Low-molecule-weight silicon oil.

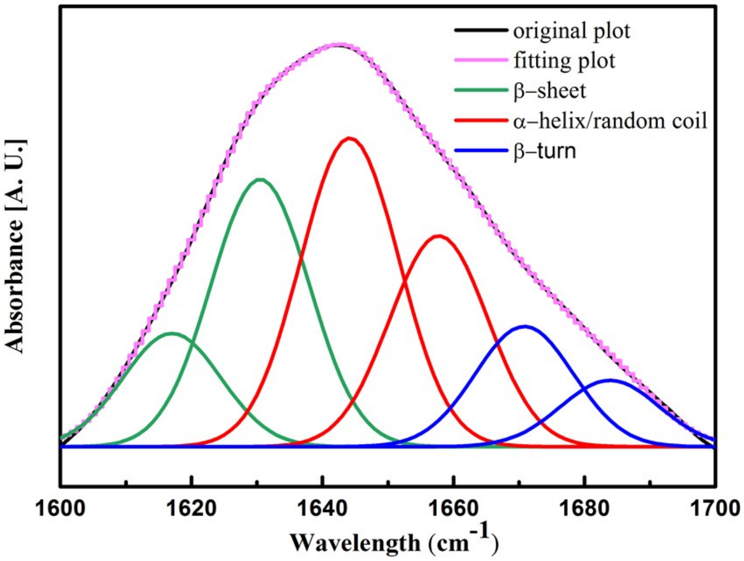

Figure S3. Selected ATR-FTIR spectra of original silk spheres deduced after Fourier self-deconvolution. Within amide I band, the contributions of β-sheets, random coil/ α-helix and β-turn are represented.

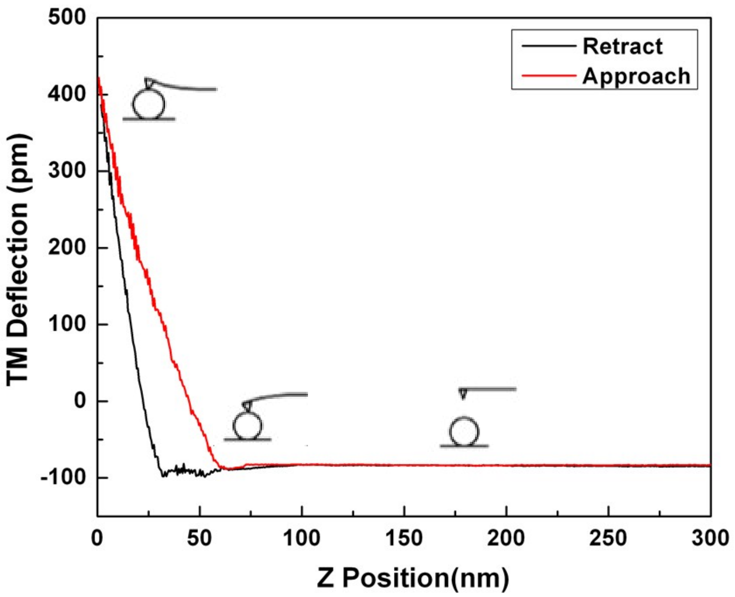

Figure S4. Nanoindentation curve of a single silk sphere.

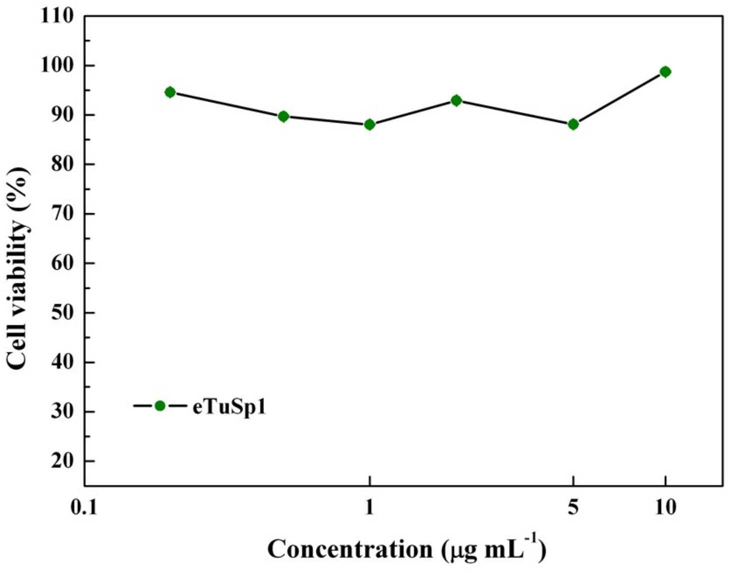

Figure S5. Cytotoxicity of pure eTuSp1 spheres.

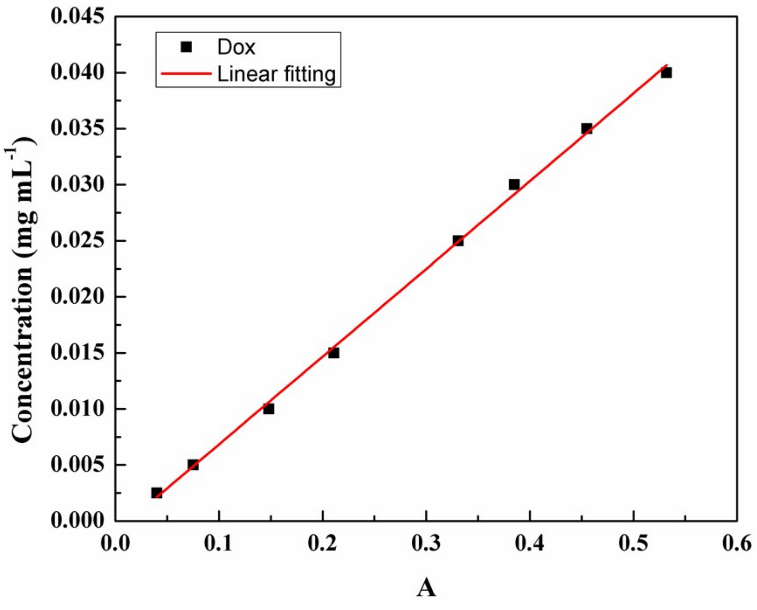

Figure S6. The standard concentration plot of the Dox. The absorbance of each sample is measured at the wavelength of 490 nm by UV-Vis spectrometry.

Table S1. Physicochemical properties of eTuSp1 spheres.

	Size (nm)	Polydispersity index	Zeta potential (mV)	Mobility ($cm^2/Vs \pm SD$)
eTuSp1	183	0.214	-33.5 ± 4.2	$-2.48\times10^{4} \pm 2.14\times10^{5}$
